# Editorial: Fluid overload in the critically ill

**DOI:** 10.3389/fmed.2023.1166202

**Published:** 2023-03-03

**Authors:** Tine S. Meyhoff, Sine Wichmann, Anna S. Messmer

**Affiliations:** ^1^Department of Intensive Care, Rigshospitalet, University of Copenhagen, Copenhagen, Denmark; ^2^Collaboration for Research in Intensive Care (CRIC), Copenhagen, Denmark; ^3^Department of Anaesthesia and Intensive Care, Nordsjællands Hospital, University of Copenhagen, Copenhagen, Denmark; ^4^Department of Intensive Care Medicine, Inselspital, Bern University Hospital, University of Bern, Bern, Switzerland

**Keywords:** fluid overload, de-resuscitation, sepsis, heart failure, trauma, fluid management

Fluid overload in the critically ill has been a clinical concern and a topical research question in the past decades. Numerous investigations formed in the wake of two landmark trials where higher intravenous fluid volumes led to worse outcome (1, 2). The first trial enrolled adults with acute lung injury (1), whereas the latter included African children with severe infection (2). Subsequent studies in broader populations of critically ill patients have added to these findings (3, 4), and in recent years, trials on enhanced fluid strategies have emerged globally (5–7). This has challenged conventional fluid management and raised additional questions. In this Research Topic, we encouraged contributions on the various aspects of fluid overload in the critically ill.

The Research Topic comprises six papers covering early fluid resuscitation and vasopressors, fluid management in sub-populations of critically ill patients, fluid overload assessment, and de-resuscitative strategies from clinicians' perspectives.

In a narrative review, Macdonald et al. provide an overview of contemporary evidence for fluids vs. vasopressors in the early resuscitation of sepsis and septic shock. The review outlines theories for sepsis pathogenesis, including micro- and macro-circulatory alterations, and the potential hemodynamic interactions of intravenous fluids and vasopressors. Ultimately, the rationale for a trial of restricted fluids and early vasopressors in septic shock is outlined.

Fluid overload has no uniform definition, diagnostic test, or management guideline in the general critically ill population. Zeuthen et al. report the results of an international survey among 1,066 Intensive Care Unit (ICU) physicians on the assessment and treatment of fluid overload. It describes current de-resuscitative strategies in the ICU, including that most clinicians supported a weight-based definition of fluid overload (minimum 5-10% increase in body weight), and most considered fluid overload a modifiable risk for morbidity. The most frequent choice of treatment was diuretics, followed by fluid restriction.

Patients with heart failure present a particular challenge for fluid management. Lower blood pressures *via* afterload reduction may have unloading benefits, but low preload and low blood pressures may also reduce coronary flow. Thus, hemodynamic changes can be both beneficial and detrimental in this patient population. As patients with heart failure often require fluids to improve cardiac output in the initial phase, they may represent a population who is specifically prone to to fluid overload at a later stage. In a retrospective cohort study, Dong et al. evaluated the association between fluid management and in-hospital mortality in ICU patients with sepsis and heart failure. The authors propose *fluid accumulation index (fluid balance/fluid intake ratio)* as a tool to assess fluid overload, as the fluid accumulation index was found to be a risk factor for in-hospital mortality in these patients.

Waskowski et al. included 2,158 patients admitted to ICU due to severe heart failure and/or cardiogenic shock in a retrospective cohort study. In contrast to other studies, no association between fluid overload (>5% increase in body weight) at ICU discharge and 30-day mortality was found in this population. Notably, <10% of the study population had fluid overload at ICU discharge, indicating that most of the population was already de-resuscitated at that time. In this study, open-heart surgery prior to admission, prior liver disease, and lactate at baseline were associated with fluid overload at ICU discharge, while disease severity or pre-existing kidney disease were not.

While the bulk of fluid research has focused on early intrahospital management, Jensen et al. investigated the effect of prehospital transportation on fluid administration in the first 24 h in patients with suspected infection. In this *post-hoc* analysis of a prospective observational study, the authors found that patients transported with emergency medical services received more fluid than those arriving at the hospital on their own, after adjustment for confounders. Importantly, the majority of the study population had simple infection or sepsis, and these patients are not directly represented in the Surviving Sepsis Guideline (8). Pre-hospital treatment might be worth considering in future guidelines updates.

Trauma patients have a unique pathophysiology, often characterized by hypovolemic shock followed by systemic inflammation, thus representing a separate sub-population of critically ill patients. In a retrospective study of 52 trauma patients, Wrzosek et al. found that infused fluids and fluid balance on day 2 of ICU stay were associated with increased mortality. Fluid balances and fluid input were correlated with the number of blood products infused, as well as with coagulation parameters, suggesting that hemorrhagic shock might have been a driver of excess fluid administration and poor outcome in this study.

In summary, this Research Topic covers some of the key aspects of fluid overload in the critically ill and addresses specific populations and time points, where fluid management could impact the patient's trajectory (Figure 1). We emphasize, that bias arising from e.g., confounding by indication and time-dependent exposure challenges the interpretation of the observational studies included in this Topic, and they inherently risk overestimating treatment effects (9). However, the studies underline clinical uncertainties in both definition and treatment of fluid overload, which warrants high-quality trials in these aspects.

**Figure 1:**
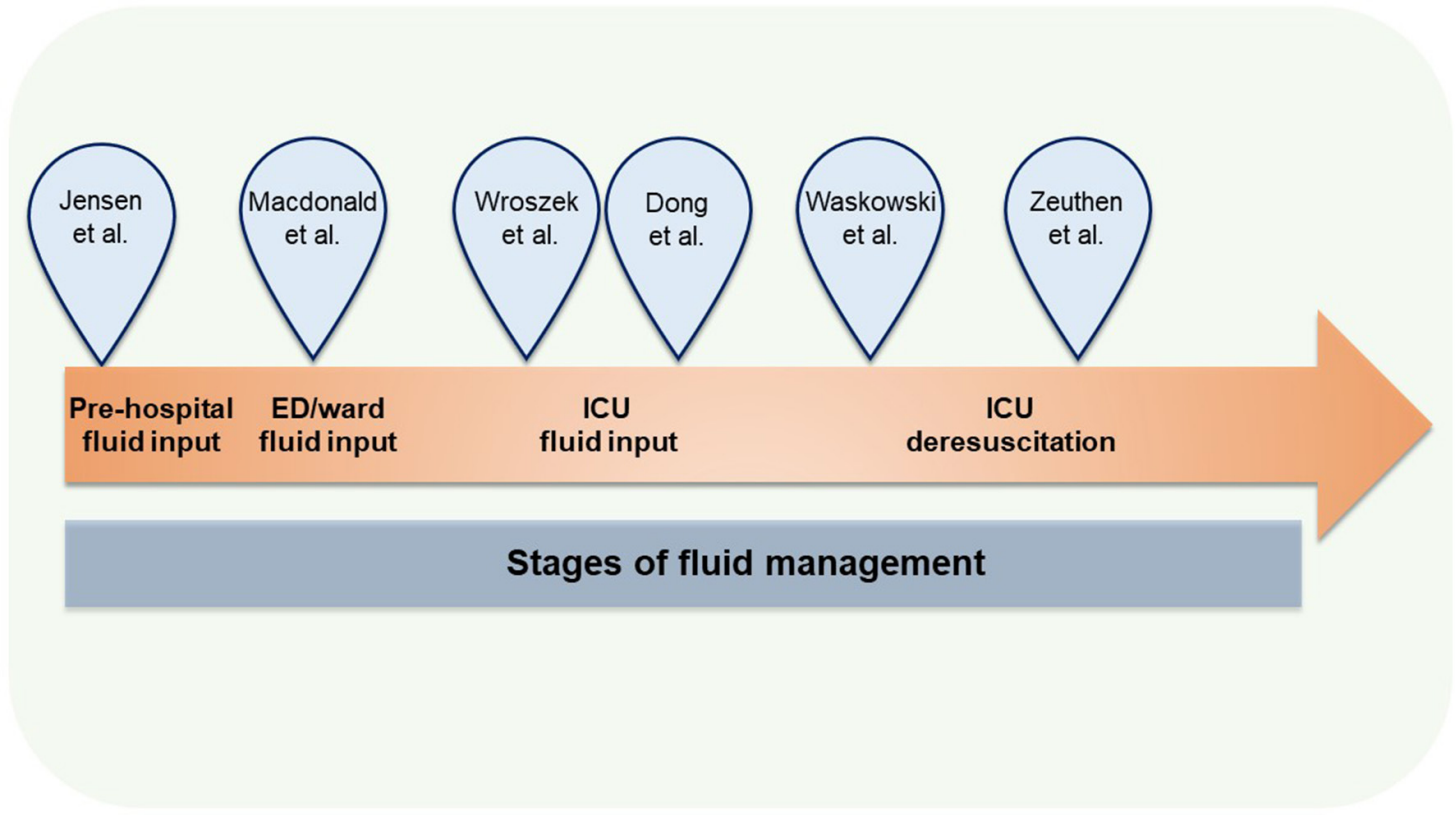
Studies in the Research Topic according to focus areas. ED, Emergency department; ICU, Intensive Care Unit.

## Author contributions

TM, SW, and AM co-wrote the manuscript. All authors contributed to the article and approved the submitted version.
